# Editorial: Applications of optical coherence tomography angiography (OCTA) in ocular complications of diabetes mellitus

**DOI:** 10.3389/fendo.2025.1743554

**Published:** 2026-01-07

**Authors:** Kaveh Fadakar, Pasha Anvari, Reza Mirshahi

**Affiliations:** 1 Eye Research Center, The Five Sense Health Institute, Iran University of Medical Sciences/Moheb Kowsar Hospital, Tehran, Iran; 2 Department of Ophthalmology, Feinberg School of Medicine, Northwestern University, Chicago, IL, United States

**Keywords:** artificial inteligence-ai, diabetic macular edema (DME), diabetic retinopathy, OCTA, optical coherence tomogmphy angiography

Diabetic retinopathy (DR) is a significant complication of diabetes mellitus (DM) that poses a great risk of irreversible vision loss. Microvasculopathy progresses gradually, leading to severe retinal ischemia in advanced stages. Excessive vascular endothelial growth factor (VEGF) production in response to ischemia causes the formation of aberrant neovessels alongside the vitreoretinal interface (1). These are the main precursors of future complications such as tractional retinal detachment or vitreous hemorrhage. Additionally, disruption of the retinal-blood barrier leads to diabetic macular edema (DME), another significant cause of vision impairment (2).

Optical coherence tomography angiography (OCTA) provides a depth-resolved, non-invasive imaging of the retinal vasculature. This advancement has revolutionized our understanding of the retinal microvascular disease. OCTA has the ability to help clinicians preventing vision-threatening complications of DR. It includes applicability in DR screening, staging, predicting future vision loss, and providing prognostic features for DME. (3, 4). In this Research topic, we compiled representative examples of the key applications of OCTA in diabetic retinopathy.

Some evidence indicates that macular OCTA imaging parameters correlate with the severity of DR. As the disease progresses, a higher degree of capillary density formation occurs in the retinal capillary layers. This has also been observed in the peripapillary retinal nerve fiber layer (pRNFL), where the average pRNFL thickness and vascular density are reduced in diabetic patients, especially those with non-proliferative DR (NPDR), compared to age-matched healthy individuals. pRNFL capillary density has a relatively high ability to distinguish DR from healthy eyes (Cao et al.). Recent studies investigating the progression of DR have highlighted the vital predictive role of peripheral non-perfusion in future complications. Ultrawide-field fluorescein angiography is a diagnostic tool that provides valuable insights into the degree of retinal ischemia. However, it is invasive and has limited access in routine ophthalmology clinics. Guo et al. showed that ultrawide-field OCTA reveals non-perfusion areas within its field of view, underscoring its potential role as a surrogate for invasive diagnostic procedures (Bi et al.). More extensive capillary nonperfusion in both the superficial and deep retinal capillary layers has been observed in eyes with DME. The current finding of reduced capillary vascular density and altered choriocapillaris flow area accentuates the importance of retinal ischemia in DME development (Cui et al.). Future longitudinal investigations utilizing OCTA parameters can help predict which eyes are at risk for DME and provide guidance for devising prophylactic plans.

Understanding retinal vascular density could expand our knowledge of certain disease processes. Corneal microdots are highly reflective, spherical structures found throughout the entire depth of the cornea. OCTA could shed light on the mechanisms of microdot development. Low-grade inflammation and relative ischemia could be the initiating factors, as these microdots appear more frequently in corneas with diabetic retinopathy, and their prevalence correlates with OCTA parameters. (Wang et al.).

The advent of artificial intelligence heralds future advancements in the care of patients with DR. Herein, we included a meta-analysis reporting the accuracy of deep learning models in diagnosing DR using OCT and retinal imaging (Guo et al.). These models showed promising performance; however, significant challenges remain to be addressed. Generating standardized imaging datasets is a crucial infrastructural issue. It is of paramount importance to pay attention to including high-quality OCTA images, with accepted image processing techniques, and to develop a platform to uniformly compare data from various devices to ensure high-quality input for deep learning models. Subtle ocular characteristics should be considered in the accurate reporting of OCTA images, including axial length, refractive error, or even mydriasis (Zhang et al.). Although the presence of mydriasis did not affect vascular density measurement in images with a comparable quality score, this simple intervention can significantly affect overall image quality in a retinal clinic.

The current Research topic explores diverse aspects of OCTA application in DR, shedding light on the vast applicability of this imaging modality. Future investigations will further demonstrate the utility of OCTA as a non-invasive and accessible imaging modality for DR patients.
